# Rapid Degradation of Bisphenol F Using Magnetically Separable Bimetallic Biochar Composite Activated by Peroxymonosulfate

**DOI:** 10.3390/molecules29235545

**Published:** 2024-11-24

**Authors:** Hui Liang, Ruijuan Li, Tongjin Liu, Rumei Li, Yuxiao Zhu, Feng Fang

**Affiliations:** 1 Institute of Plant Protection, Shandong Academy of Agricultural Sciences, Jinan 250100, China; huiliangchem@163.com (H.L.); ruijuanli@163.com (R.L.); tongjinliu2024@163.com (T.L.); rumeili0815@163.com (R.L.); 2 Shandong Key Laboratory for Green Prevention and Control of Agricultural Pests, Jinan 250100, China

**Keywords:** carbon-based catalysts, peroxymonosulfate, rapid degradation, magnetic separation, organic pollutants

## Abstract

Peroxymonosulfate (PMS)-based advanced oxidation processes have shown potential for the removal of organic contaminants; however, the preparation of catalysts with high degradation efficiencies and rapid reaction rates remains a challenge. In this study, we have successfully synthesized CoFe bimetallic modified corn cob-derived biochar (CoFe/BC) for the activation of PMS, achieving the rapid and efficient degradation of bisphenol F (BPF). The synthesized CoFe/BC catalyst demonstrated excellent catalytic performance, achieving over 99% removal within 3 min and exhibiting a removal rate of 90.0% after five cycles. This could be attributed to the cyclic transformation of Co and Fe, which sustained rapid PMS activation for BPF degradation, and Co^0^/Fe^0^ played a significant role in the cyclic transformation. Furthermore, the electron paramagnetic resonance tests confirmed that •SO_4_^−^ and •OH were the primary reactive oxygen species, while •O_2_^−^ played a minor role in BPF degradation. This study highlights the high degradation efficiency, rapid reaction rate, excellent magnetic separation properties, and exceptional reusability of CoFe/BC catalysts for BPF removal, providing valuable insights for practical wastewater treatment.

## 1. Introduction

As a replacement for bisphenol A, bisphenol F (BPF) has been widely used in various industrial applications. Owing to its high water solubility, BPF has abundantly accumulated in lakes, rivers, and drinking water, threatening the environment and human health [1,2,3]. Therefore, it is critical to develop efficient methods for BPF removal.

Advanced oxidation processes (AOPs), an effective technology for the degradation of organic contaminants, have received increasing attention for BPF removal [4,5,6]. Among the various AOPs, peroxymonosulfate (PMS)-based AOPs have recently garnered significant interest owing to the mild reaction conditions of PMS, as well as its high selectivity and ability to degrade pollutants thoroughly [7,8]. Transition metals can efficiently activate PMS to generate active species with strong oxidizing abilities for pollutant degradation [9,10,11]. Zou et al. prepared a novel N, O dual-defect co-modified 0D/2D hybrid of CuO nanodots (NDs)/g-C_3_N_4_ nanosheets and used it for tetracycline degradation, and it exhibited excellent catalytic activity owing to the generation of surface high-valent copper species [12]. Zhao et al. proved that 88% of diniconazole was removed in an α-MnO_2_/PMS system, which was four times higher than that in only PMS, and the degradation of diniconazole was affected by acidic conditions and co-existing anions [13]. Zhang et al. demonstrated that a Co-CN catalyst with CoN_x_ sites highly dispersed on a porous carbon nitride displayed outstanding organic contaminant degradation ability and an extremely fast catalytic rate of 0.194 min^−1^ [14]. Additionally, active species (including •SO_4_^−^, •OH, •O_2_^−^, and ^1^O_2_) and electron transfer played an important role in the tetracycline degradation. Transition metals such as Fe, Ti, Ni, and Mo are commonly used as catalysts for peroxymonosulfate activation [15,16,17,18]. Among these transition metals, Fe, which is relatively nontoxic and cost-effective, and Co exhibit the highest catalytic performance in activating PMS and are highly promising candidate catalysts.

As outstanding carrier materials, carbonaceous materials can prevent the aggregation of transition metal particles, enhance electron transfer, and reduce the leaching of metal ions [19,20,21]. Therefore, composites of carbonaceous materials and transition metal oxides exhibit a better PMS activation for the removal of organic pollutants. For instance, CoFe spinel oxide loaded on the surface of porous biochar achieved a 99% removal of 4-nitrophenol in 10 min, attributed to well-dispersed spinel, enriched oxygen vacancies, and pyrrole N for active species (•SO_4_^−^, •OH, and ^1^O_2_) formation [22]. Kaur found that cotton shell-derived activated carbon immobilized with Fe^0^ (ZVI@CSAC) showed a notable performance in PMS utilization and inherent stabilities, and the removal of rhodamine B and crystal violet reached 87.6% and 99.8%, respectively. Moreover, they demonstrated that the synergistic interaction between the adsorption and activation processes enhanced the overall efficiency of dye degradation [23]. Additionally, Nguyen et al. prepared a flower-like core–shell CNBS@MnO_2_ heterojunction and used it for diclofenac degradation in the presence of visible light and PMS. The catalyst displayed an enhanced photodegradation performance in activating PMS after 15 min of irradiation, which was due to the large surface area, reduced interfacial resistance for rapid electron transport, and •SO_4_^−^ constantly generated by the activation of PMS through the redox cycling of Mn species [24]. Mi et al. showed that the valence cycles of Cu^+^/Cu^2+^ could promote PMS activation to effectively degrade tetracycline. Notably, the valence cycles of Cu^+^/Cu^2+^ are caused by the complexation of tetracycline with the Cu sites of the catalyst, leading to the rearrangement of active sites to easily obtain electrons, accelerating the reduction of Cu^2+^ to Cu^+^ [25]. Despite the aforementioned advances, the rapid promotion of PMS activation for the removal of pollutants from water remains challenging.

In this study, CoFe bimetallics were anchored to corn cob-derived biochar (BC) to remove BPF in the presence of PMS. The produced CoFe/BC catalyst exhibited a high efficiency for PMS activation, and the BPF removal rate could exceed 99% in 3 min. The CoFe/BC catalyst exhibited excellent reusability, with a removal rate of 90.0% after five cycles. Additionally, a radical pathway (especially •SO_4_^−^ and •OH) degradation mechanism was proposed, and the high degradation efficiency, rapid reaction rate, outstanding magnetic separation properties, and exceptional reusability of the CoFe/BC catalyst were promising for future applications.

## 2. Results

**Figure 1:**
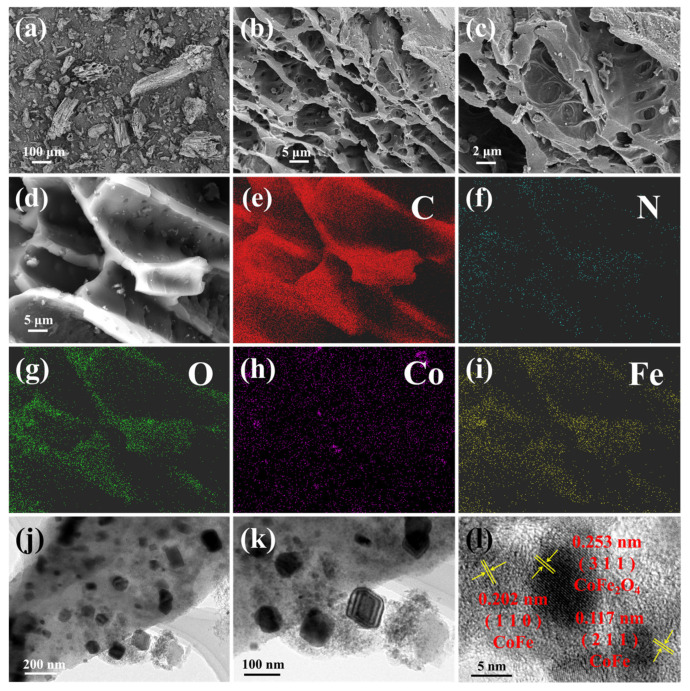
(**a**–**d**) SEM images, (**e**–**i**) EDS mappings (C, N, O, Co, Fe), and (**j**–**l**) HRTEM images of CoFe/BC catalyst.

The structure, morphology, and elemental distribution of the as-prepared catalyst CoFe/BC were revealed using SEM and HRTEM; the results are shown in Figure 1. The composites had an irregular morphology with uneven grain sizes, and all the particles displayed an open tubular structure with a thickness of approximately 1 μm and a tube diameter of 5–20 μm (Figure 1a,b). Moreover, many pores of varying sizes were distributed on the tube wall and did not penetrate the wall to connect with each other. This typical tubular structure with hierarchical pores exposes more active catalytic sites for the adsorption and degradation of pollutants [26]. As shown in the enlarged images (Figure 1c,d), many small particles of tens of nanometers attached to the surface or dispersed in the pores of the CoFe/BC catalysts were also found. Figure 1e–i shows the SEM-EDS elemental mapping results, which clearly demonstrate that C, N, O, Co, and Fe were uniformly distributed throughout the catalyst, proving the successful doping of Co and Fe into BC. Additionally, from the results of the energy-dispersive spectroscopy (EDS) analysis of CoFe/BC (Figure S1), it can be seen that the contents of Co and Fe were approximately 4.26% and 3.65%, respectively, and this was obviously different from the amounts added during synthesis, which was caused by the incomplete loading of metal ions during the catalyst preparation process.

The morphologies of the CoFe/BC catalyst were further verified using HRTEM, revealing the presence of numerous particles several tens of nanometers in size (Figure 1j,k), which is consistent with the SEM results. However, tubular structured particles were not observed, likely due to breakage during the pre-test treatment. High-magnification TEM images (Figure 1l) indicated that abundant lattices were distributed within the CoFe/BC. The lattice spacings of 0.202 and 0.117 nm corresponded well with the (110) and (211) planes of CoFe, respectively. Additionally, the lattice spacing of 0.253 nm was associated with the (311) crystal planes of CoFe_2_O_4_, suggesting the presence of CoFe_2_O_4_ in the CoFe/BC catalyst.

**Figure 2:**
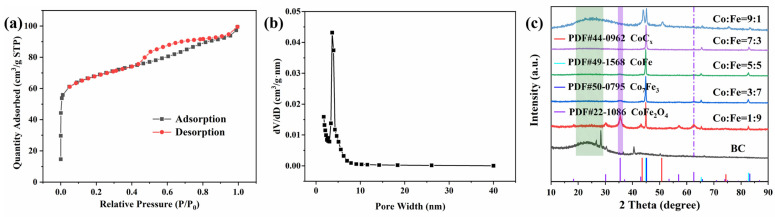
(**a**) N_2_ adsorption–desorption isothermal curve and (**b**) pore-size distribution curve of CoFe/BC catalyst; (**c**) XRD patterns of BC, CoFe_2_O_4_/CoFe/BC (Co:Fe = 1:9), CoFe/BC37 (Co:Fe = 3:7), CoFe/BC (Co:Fe = 5:5), CoFe/BC73 (Co:Fe = 7:3), and CoC_x_/Co_7_Fe_3_/BC (Co:Fe = 9:1) catalysts. The proportion of Co and Fe mentioned in this study is the molar ratio, and further information on the catalysts is available in Section 3.2. Synthesis.

Large specific surface areas and abundant pore structures are beneficial for pollutant adsorption and the degradation of pollutants [27]. For further confirmation of its surface properties, the specific surface area and pore-size distribution of CoFe/BC were characterized using N_2_ adsorption–desorption measurements. As shown in Figure 2a, CoFe/BC exhibited type IV isotherms with H4 hysteresis loops, indicating the presence of a mesoporous structure [28]. The specific surface area of CoFe/BC was able to reach 254.26 m^2^/g, with an average diameter of 4.13 nm (Figure 2b). Furthermore, other catalysts prepared with different Co/Fe ratios were also characterized by N_2_ adsorption–desorption isotherm (Figure S2), and the BET surface area and the average pore size are shown in Table S1. Generally, CoFe/BC exhibits a highest specific surface area and superior pore structure, which effectively disperses CoFe nanoparticles and exposes more catalytic sites.

The crystal structures of the catalysts were determined using XRD. In the XRD pattern of the BC in Figure 2c, the broad peak at 23.7° corresponds to the characteristic peak of the (002) crystal plane of poorly graphitic carbon, as previously reported [29]. Additionally, impurity peaks were observed in the BC, indicating the presence of impurities in the BC, which were attributed to the inorganic salts in corn cobs.

When Co and Fe were introduced into the BC, the diffraction peak of CoFe_2_O_4_/CoFe/BC matched well with that of CoFe_2_O_4_ (JCPDS No. 22-1086), and the peaks at 44.87°, 65.31°, and 82.74° corresponded to the (110), (200), and (211) crystal planes of standard CoFe (JCPDS No. 49-1568), respectively. As the proportion of Co increased, the peak intensity and area of the two catalysts (CoFe/BC37 and CoFe/BC) at 2θ = 35.44° and 62.59° (corresponding to CoFe_2_O_4_) showed a tendency to decrease, which was not observed in CoFe/BC73, implying that the CoFe_2_O_4_ structure was gradually decreasing until it completely disappeared. Furthermore, the (002) crystal planes of carbon were observed in CoFe_2_O_4_/CoFe/BC, CoFe/BC37, CoFe/BC, and CoFe/BC73 (Figure S3). New peaks attributed to CoC_x_ (JCPDS No. 44-0962) were observed for CoC_x_/Co_7_Fe_3_/BC, and the diffraction peaks at 45.17°, 65.71°, and 83.25° matched those of Co_7_Fe_3_ (JCPDS No. 50-0795) [30,31].

**Figure 3:**
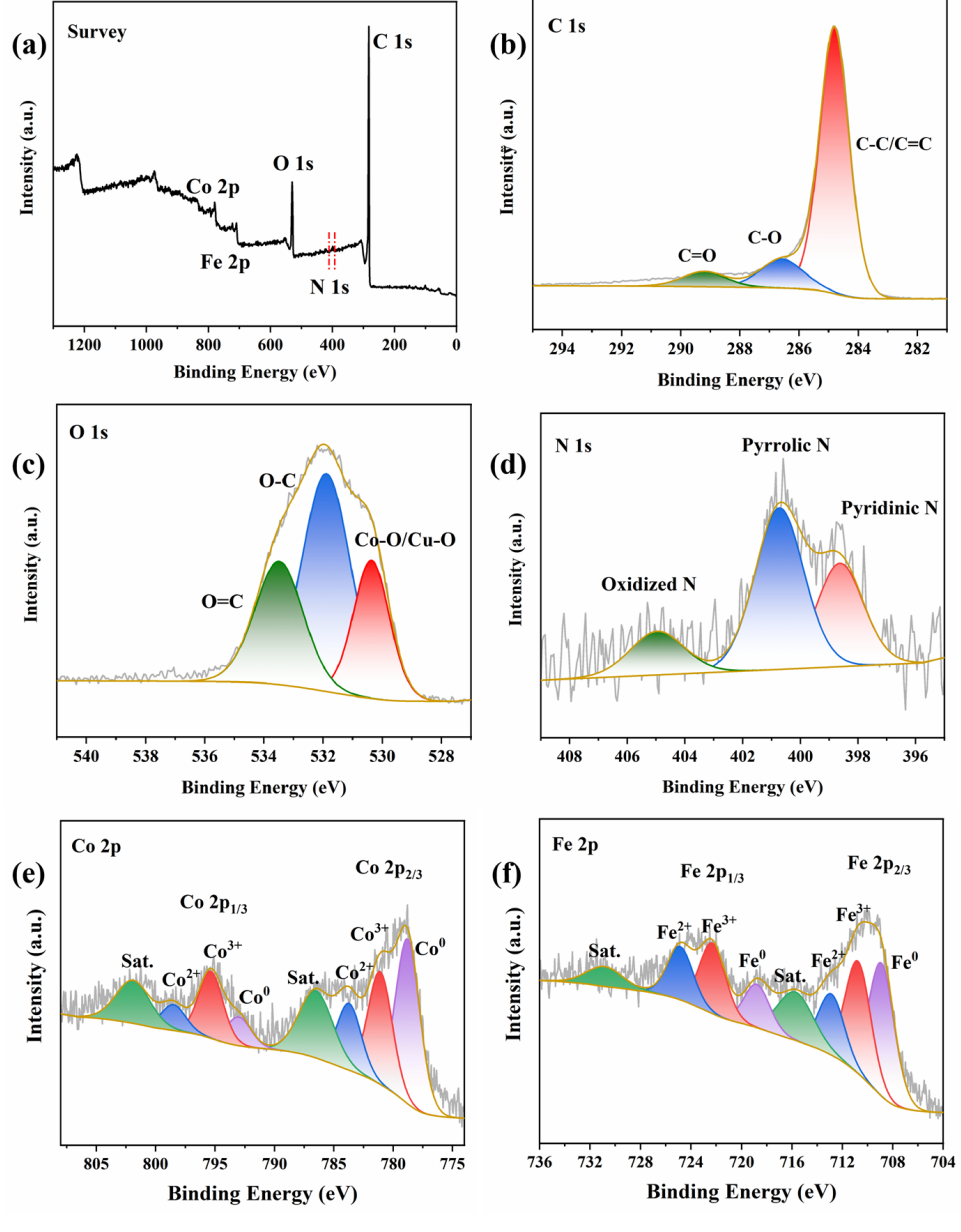
(**a**) XPS survey, (**b**) C 1s, (**c**) O 1s, (**d**) N 1s, (**e**) Co 2p, and (**f**) Fe 2p of CoFe/BC catalyst.

To investigate the element composition and valence band structure, the CoFe/BC catalyst was analyzed using XPS measurements. Figure 3a shows the C 1s, O 1s, N 1s, Co 2p, and Fe 2p peaks in the XPS survey spectrum. The fine C 1s spectrum displayed three peaks corresponding to C-C/C=C, C-O, and C=O [32], respectively. Figure 3c shows the high-resolution XPS O 1s spectra, which were deconvoluted into three oxygen species: lattice oxygen (Co-O/Fe-O, 530.36 eV), O-C (531.89 eV), and O=C (533.48 eV) [33]. Furthermore, the N 1s spectrum revealed three peaks attributed to pyridinic N (398.63 eV), pyrrolic N (400.73 eV), and oxidized N (404.96 eV) [34,35].

As shown in Figure 3e, the Co 2p XPS pattern exhibited peaks at 778.78 eV and 792.99 eV belonging to Co^0^, two peaks at 781.02 eV and 783.76 eV in the Co 2p_3/2_ spectrum, and two peaks at 795.35 and 798.49 eV in the Co 2p_1/2_ spectrum, which corresponded to Co^3+^ and Co^2+^; two additional satellite peaks at 786.51 eV and 801.92 eV were also observed [32,36]. Moreover, as shown in Figure 3f, the high-resolution Fe 2p spectra were fitted with six peaks for Fe^0^ (708.90 eV and 718.79 eV), Fe^3+^ (710.76 eV and 722.29 eV), and Fe^2+^ (712.88 eV and 724.85 eV), with additional peaks (715.73 eV and 730.85 eV) representing satellite vibrations [37].

**Figure 4:**
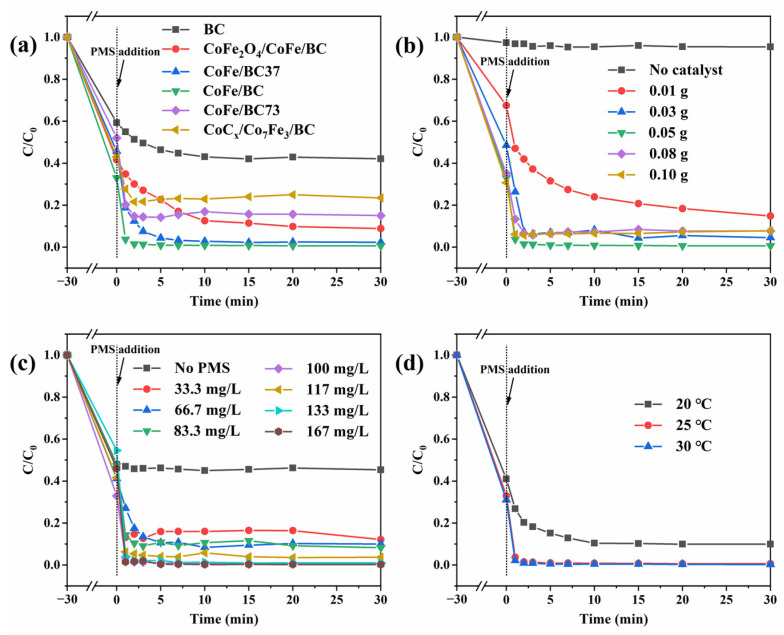
(**a**) Degradation efficiencies of BPF degradation on different catalysts. Influence of various conditions on BPF degradation in CoFe/BC/PMS system: (**b**) catalyst amount, (**c**) PMS concentration, and (**d**) reaction temperature. Experimental conditions: Catalyst = 0.05 g, BPF = 10 mg/L, PMS = 100 mg/L, T = 25 °C.

The catalytic activity of CoFe/BC for BPF removal via PMS activation is shown in Figure 4a. For comparison, BPF removal was also assessed using BC, CoFe_2_O_4_/CoFe/BC, CoFe/BC37, CoFe/BC73, and CoC_x_/Co_7_Fe_3_/BC. All catalysts exhibited an excellent adsorption capacity for BPF, and the removal rates exceeded 40% within 30 min, which might be attributed to the large specific surface area and abundant pore structure of the catalysts. As shown in Figure 4a, the BPF adsorption in the presence of BC reached 40.8% after 30 min, and an additional 17.1% of BPF was removed after the introduction of PMS, indicating the activation ability of the BC catalyst for PMS, which is consistent with previous studies [38,39].

When Co and Fe were introduced into the system, the BPF rapidly degraded after 30 min of pre-adsorption. Compared with BC, BPF removal was 91.1%, 85.0%, and 76.6% over CoFe_2_O_4_/CoFe/BC, CoFe/BC73 and CoC_x_/Co_7_Fe_3_/BC, respectively. The BPF removal rate increased to 97.7% in the presence of CoFe/BC37. Considering the adsorption ability of CoFe/BC, adsorption experiments were conducted to clarify the role of the catalytic process during BPF removal (Figure 4c, the No PMS curve), and the BPF adsorption in the presence of CoFe/BC was 54.7%. Moreover, it is worth noting that a high efficiency in BPF removal (>99%) was achieved in 3 min with the catalyst CoFe/BC, and the corresponding degradation rate k was 1.0657 min^−1^, which is 49.6, 9.1, 3.0, 1.7, and 3.1 times higher than that of BC, CoFe_2_O_4_/CoFe/BC, CoFe/BC37, CoFe/BC73, and CoC_x_/Co_7_Fe_3_/BC (Figure S4), respectively. As displayed in Figure 4b, approximately 5% BPF was removed using only PMS, implying the low BPF degradation efficiency of PMS [40]. Remarkably, BPF removal increased in the presence of both CoFe/BC and PMS. The amount of catalyst increased from 0.01 to 0.10 g, and the BPF removal rate initially increased and then decreased slightly. This phenomenon might be attributed to the reaction of reducing substances (including Fe^0^, Co^0^, Fe^2+^, and Co^2+^) with the generated •SO_4_^−^ in the CoFe/BC/PMS system, inhibiting the oxidation of BPF by •SO_4_^−^ [41].

Excessive PMS has been reported to generate competitive adsorption with pollutants, thereby reducing degradation efficiency [42]. However, in this study, the BPF removal rate gradually increased with an increasing PMS dosage. This can be attributed to the large specific surface area and numerous active sites of the CoFe/BC catalyst, making it unlikely for PMS to compete with BPF for adsorption. Additionally, the removal efficiency improved with an increasing temperature (Figure 4d), indicating that the degradation efficiency was positively correlated with temperature. This is because the reaction temperature not only enhanced the PMS activation efficiency but also reduced the reaction barrier, accelerating the decomposition of pollutants [43,44]. Considering both degradation effectiveness and economic efficiency, a catalyst dosage of 0.05 g and PMS concentration of 100 mg/L were selected for further experiments.

**Figure 5:**
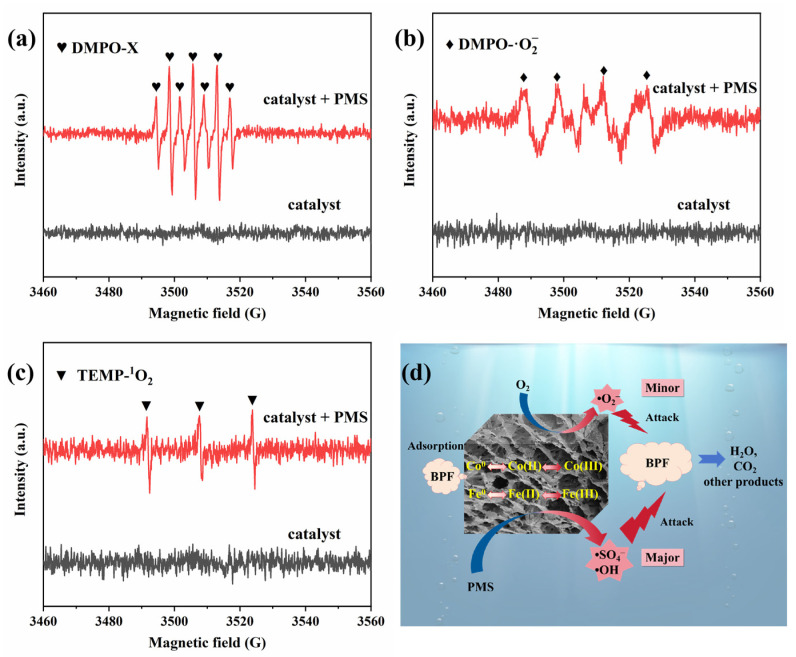
EPR spectra of (**a**) DMPO-X, (**b**) DMPO-•O_2_^−^, and (**c**) TEMP-^1^O_2_; (**d**) the proposed mechanism for BPF degradation over the CoFe/BC/PMS system.

Electron paramagnetic resonance (EPR) measurements were used to determine the active species in the degradation process, using DMPO and TEMP as trapping agents. The EPR signals of DMPO-•SO_4_^−^, DMPO-•OH, DMPO-•O_2_^−^, and TEMP-^1^O_2_ were essentially undetectable in the reaction system without PMS (Figure 5a–c), confirming that •SO_4_^−^, •OH, •O_2_^−^, and ^1^O_2_ were not generated at this stage. As depicted in Figure 5a, no expected characteristic signal peaks of DMPO-•SO_4_^−^ and DMPO-•OH were observed in the CoFe/BC/PMS system. Instead, an exceptionally strong signal with peak intensities of 1:2:1:2:1:2:1 for DMPO-X was captured, which was the direct oxidation product of DMPO by the reaction species generated from PMS activation [45,46]. This indicates that amounts of active species (•SO_4_^−^ and •OH) with a strongly oxidizing capacity were produced with the activation of PMS by CoFe/BC [47]. Additionally, the signals of DMPO-•O_2_^−^ were detected in the presence of both CoFe/BC and PMS (Figure 5b), implying that •O_2_^−^ indeed participated in the BPF degradation process. The triplet signal of TEMP-^1^O_2_ disappeared to a great extent in the CoFe/BC/PMS system (Figure 5c), probably because of the limited generation of ^1^O_2_ radicals. Generally, •SO_4_^−^, •OH, and •O_2_^−^ contributed to the degradation of BPF and •SO_4_^−^, •OH, playing a dominant role in the removal of BPF in the CoFe/BC/PMS system.

Based on the results, a possible mechanism for BPF removal is proposed (Figure 5d). In the CoFe/BC/PMS system, BPF is degraded through a radical pathway, with •SO_4_^−^ and •OH serving as the primary active species. First, owing to the large specific surface area and abundant pore structure of CoFe/BC, a great amount of contaminants were adsorbed onto the surface of the catalyst during pre-adsorption for further degradation. Subsequently, with the introduction of PMS, a significant amount of •SO_4_^−^ was primarily produced through the transfer of electrons between Co^0^/Co^2+^ (or Fe^0^/Fe^2+^) and PMS (Equations (1) and (2)) [36,48]. A portion of the freshly formed •SO_4_^−^ is further transformed into •OH (Equation (3)) [49]: accompanying the electron transfer, the dissolved oxygen in water would acquire electrons to partially generate •O_2_^−^ (Equation (4) [20]. Furthermore, Co^3+^/Fe^3+^ is reduced to Co^2+^/Fe^2+^ by Co^0^/Fe^0^ (Equation (5)), which could then provide electrons to the PMS for the generation of •SO_4_^−^. The literature indicates that high-valent Co/Fe captures electrons from PMS to form low-valent Co/Fe [50], maintaining a high catalytic efficiency. The•SO_5_^−^ generated along with this reaction will further decompose to yield ^1^O_2_ [51], which was nearly undetectable in the CoFe/BC/PMS system. Therefore, Co^0^/Fe^0^ possibly plays a significant role in the reduction of Co^3+^/Fe^3+^, and the redox cycles of high-valent Co/Fe and low-valent Co/Fe facilitate the continuous activation of PMS. Finally, the produced reactive oxygen species, especially •SO_4_^−^ and •OH, attack the aromatic ring of BPF and degrade it into CO_2_, H_2_O, and other products (Equation (6)).

Fe^0^/Co^0^ + 2HSO_5_^−^ → Fe^2+^/Co^2+^ + 2•SO_4_^−^ + 2OH^−^ (1)

Fe^2+^/Co^2+^ + 2HSO_5_^−^ → Fe^3+^/Co^3+^ + 2•SO_4_^−^ + 2OH^−^ (2)

•SO_4_^−^ + OH− → SO_4_^2−^ + •OH (3)

O_2_ + e^−^ → •O_2_^−^ (4)

Fe^0^/Co^0^ + 2Fe^3+^/Co^3+^ → 3Fe^2+^/Co^2+^ (5)

•SO_4_^−^ + •OH + •O_2_^−^ + BPF → CO_2_ + H_2_O + other products (6)

**Figure 6:**
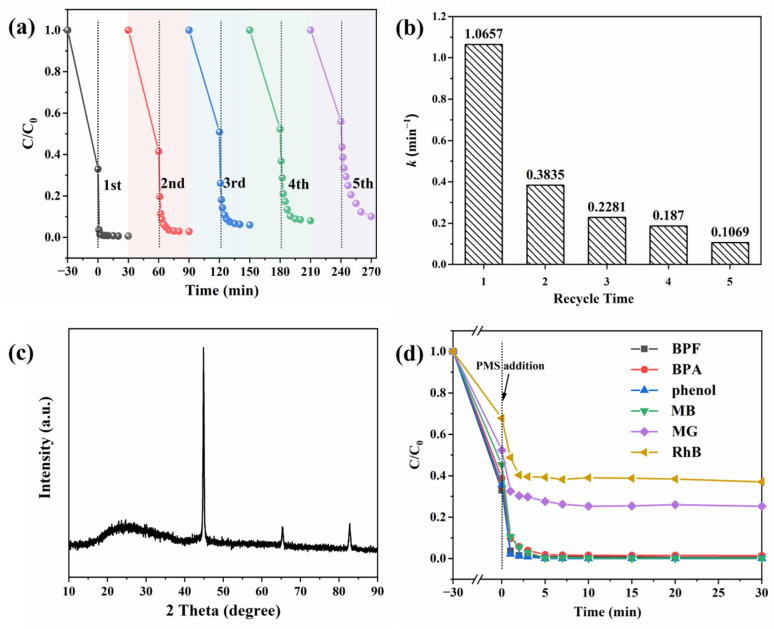
(**a**) Recyclability of CoFe/BC for BPF removal and (**b**) corresponding k value; (**c**) XRD spectrum of CoFe/BC after reaction; (**d**) degradation performance of CoFe/BC catalyst on different organic pollutants. Experimental conditions: Catalyst = 0.05 g, BPF = 10 mg/L, BPA = 10 mg/L, phenol = 10 mg/L, MB = 50 mg/L, MG = 50 mg/L, RhB = 50 mg/L, PMS = 100 mg/L, T = 25 °C.

Recovery and stability are important factors that influence the application of catalysts [52,53]. After utilization, the CoFe/BC catalyst was retrieved using a magnet and rinsed several times with ethanol and deionized water (Figure S5). The reusability and stability of the dried catalysts were then tested. As shown in Figure 6a, the BPF removal rate reduced from 99.4% to 90.0% after five cycles, and the corresponding k value decreased from 1.0657 to 0.1069 min^−1^ (Figure 6b), which might be due to the occupancy of active sites or the partial leaching of metal ions. Compared with the fresh catalyst, the peak position and strength of the catalyst after use were not significantly affected, and peaks attributed to CoFe_2_O_4_ were not observed, suggesting that the losses occurred during the recovery of CoFe/BC (Figure 6c), which might explain the reduced degradation efficiency.

To verify the catalytic ability of the CoFe/BC catalyst, we further evaluated the universality of the CoFe/BC/PMS system on the degradation of other pollutants, such as bisphenol A (BPA), phenol, methylene blue (MB), malachite green (MG) and rhodamine B (RhB). From Figure 6d, it can be seen that there was a significant degradation performance on BPA, phenol, and MB by the CoFe/BC/PMS system, and almost all the pollutants were removed within 5 min. Additionally, the removal efficiency of the CoFe/BC catalyst for MG and RhB can also reach over 60% in an extremely short period of time. These outstanding performances by the CoFe/BC catalyst are anticipated to be further employed in practice.

## 3. Materials and Methods

### 3.1. Chemicals

CoCl_2_·6H_2_O (Cobalt chloride hexahydrate, AR), FeCl_3_·6H_2_O (iron chloride hexahydrate, AR), ammonia solution (28%), and KHSO_5_·0.5KHSO_4_·0.5K_2_SO_4_ (potassium peroxymonosulfate, PMS, ≥47% KHSO_5_ basis) were provided by Macklin Biochemical Technology Co., Ltd. (Shanghai, China). Bisphenol F (BPF, 98%), bisphenol A (BPA, >99%), phenol, malachite green (MG, >95%), methylene blue (MB, AR), rhodamine B (RhB, AR), and methanol (MeOH, AR) were obtained from Aladdin Biochemical Technology Co., Ltd. (Shanghai, China). The corn cob powder was purchased online. All the chemicals were used without further treatment. Deionized water produced by the Taiping-M water system was used throughout the experiments.

### 3.2. Synthesis

Preparation of CoFe/BC composites: 1.19 g (5 mmol) cobalt chloride hexahydrate and 1.35 g (5 mmol) iron chloride hexahydrate was dissolved in 40 mL deionized water (the molar ratio of Co/Fe was 5:5). Next, 2.5 g corn cob powder was dispersed in the mixed solution and stirred for 30 min. Subsequently, 5 mL of the ammonia solution was slowly added to the reaction system with vigorous stirring for 30 min. The obtained solid product was washed with deionized water and dried at 60 °C overnight. Finally, the resulting solids were maintained at an annealing temperature of 600 °C for 3 h in a tubular furnace with a heating rate of 5 °C/min under a nitrogen atmosphere.

When the molar ratio of Co/Fe was 1:9 (Co 1 mmol, Fe 9 mmol), 3:7 (Co 3 mmol, Fe 7 mmol), 7:3 (Co 7 mmol, Fe 3 mmol), and 9:1 (Co 9 mmol, Fe 1 mmol), the as-prepared materials were labeled CoFe_2_O_4_/CoFe/BC, CoFe/BC37, CoFe/BC73, and CoC_x_/Co_7_Fe_3_/BC, respectively. The BC catalyst was synthesized by annealing corn cob powder in an N_2_ atmosphere.

### 3.3. Catalysts Characterization

Surface morphologies and detailed structures were observed using scanning electron microscopy (SEM, FEI QUANTA 250, Hillsborough, OR, USA) and transmission electron microscopy (TEM, FEI Tecnai G2 F20, Hillsborough, OR, USA). The crystal structures of the catalysts were examined using X-ray diffraction (XRD, Bruker, D8, Bremen, Germany). The specific surface area and pore-size distribution were determined using the N_2_ adsorption–desorption technique (Brunauer–Emmett–Teller (BET), Micromeritics ASAP 2460, Norcross, GA, USA). Surface elemental compositions and chemical states were investigated using X-ray photoelectron spectroscopy (XPS, Thermo Scientific K-Alpha, Waltham, MA, USA). The electron paramagnetic resonance (EPR; Bruker EMXplus-6/1, Bremen, Germany) spectra of the catalysts were obtained to investigate the reactive species generated during BPF degradation.

### 3.4. Evaluation of Catalytic Activity

Typically, pollutant degradation is conducted in a 100 mL flask. During BPF degradation, 0.05 g of the catalyst was added to a flask containing 60 mL of 10 mg/L BPF solution. The suspension was stirred for 30 min to reach equilibrium. The desired dosage of PMS (100 mg/L) was added to the system under continuous stirring to initiate BPF degradation. At preset intervals, 1 mL of the solution was withdrawn, filtered through 0.45 μm membranes, and poured into a tube containing 1 mL of methanol. The residual concentration of BPF was determined using high-performance liquid chromatography (HPLC, Waters e2695) at 230 nm, using methanol and water as the mobile phases.

Other catalytic degradation experiments were carried out under similar conditions, with only one variable being altered (for instance, catalysts, catalyst amount, PMS concentration, reaction temperature, and organic pollutants). The recycling experiments were also performed under similar conditions. The CoFe/BC catalyst, subsequent to utilization, was extracted by a magnet and washed several times with ethanol and deionized water. Then the aforementioned steps were reiterated. All the experiments were conducted three times with similar results, and the mean value is taken as the final reported result.

The concentration of other pollutants was determined by HPLC and UV–Vis spectrophotometer. Further information concerning the experiments is provided in Table S2 in the Supplementary Materials. The degradation rate (*η*) of the pollutant was calculated using the equation *η* = *C*_t_/*C*_0_ × 100%, where *C*_0_ is the initial concentration of pollutant before the reaction and *C*_t_ is the actual pollutant concentration produced at reaction time t.

## 4. Conclusions

In this study, we employed waste corn cobs as a carbon source and successfully fabricated CoFe/BC by introducing Co and Fe. The CoFe/BC catalyst demonstrated outstanding efficacy in activating PMS for BPF removal owing to its large surface area, abundant hierarchical mesopores, and high catalytic ability in activating PMS. Experimental results showed that a high efficiency of BPF removal (>99%) could be achieved in 3 min with the catalyst CoFe/BC after pre-adsorption. The removal rate of BPF continued to reached 90% after five cycles, indicating the great reusability and stability of the CoFe/BC catalyst. EPR analyses revealed that •SO_4_^−^ and •OH played the dominant roles in the degradation of BPF within the CoFe/BC/PMS system. The CoFe/BC catalyst possessed a high degradation efficiency, rapid reaction rate, outstanding magnetic separation properties, and exceptional reusability, presenting significant potential for practical environmental remediation.

## Data Availability

Data are contained within the article and Supplementary Materials.

## Supplementary Materials

The following supporting information can be downloaded at: https://www.mdpi.com/article/10.3390/molecules29235545/s1, Table S1: BET surface area and average pore diameter of prepared catalysts; Table S2: Details for analytical methods of pollutants; Figure S1: EDS analysis of CoFe/BC; Figure S2: (a) N_2_ adsorption–desorption isothermal curve and (b) pore-size distribution curve of BC, CoFe_2_O_4_/CoFe/BC, CoFe/BC37, CoFe/BC73, and CoC_x_/Co_7_Fe_3_/BC catalysts; Figure S3: XRD patterns of (a) CoFe_2_O_4_/CoFe/BC, (b) CoFe/BC37, (c) CoFe/BC, and (d) CoFe/BC73; Figure S4: (a) Degradation kinetic curves and (b) apparent rate constants k of BPF degradation in different systems. Reaction condition: Catalyst = 0.05 g, BPF = 10 mg/L, PMS = 100 mg/L, T = 25 °C; Figure S5: Photographs of the separation process of the CoFe/BC catalyst with an external magnetic field.
