# Supplementary material for: Rapid Degradation of Bisphenol F Using Magnetically Separable Bimetallic Biochar Composite Activated by Peroxymonosulfate
Source: Molecules. 2024 Nov 24;29(23):5545. doi: 10.3390/molecules29235545 (PMC11643965; doi:10.3390/molecules29235545)
Supplement: Supplementary file 1 [file molecules-29-05545-s001.zip › molecules-3305910-supplementary.pdf]

Supplementary Material

Title：Rapid degradation of bisphenol F using magnetically separable bimetallic biochar composite activated by peroxymonosulfate

Table S1. BET surface area and average pore diameter of prepared catalysts.

Catalyst	BET surface area (m^2/g)	Average diameter (nm)
BC	236.72	4.08
$CoFe_2O_4$/CoFe/BC	188.99	3.98
CoFe/BC37	232.47	3.98
CoFe/BC	254.26	4.13
CoFe/BC73	252.03	4.70
CoC_x/Co_7Fe_3/BC	246.03	5.80

Table S2. Details for analytical methods of pollutants.

Pollutantants/Analysis method	Mobile phase (V%:V%)	UV detector (nm)	Flow rate (mL/min)
BPF/HPLC	water : methanol = 30:70	230	1.0
BPA/HPLC	water : methanol = 30:70	230	1.0
Phenol/HPLC	water : methanol = 25:75	254	1.0
MB/UV-Vis		663	
MG/UV-Vis		618	
RhB/UV-Vis		554	

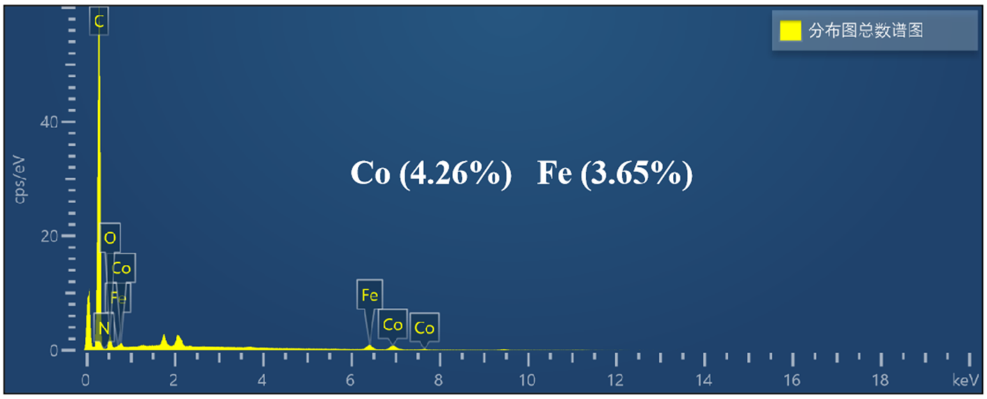

Figure S1. EDS analysis of CoFe/BC.

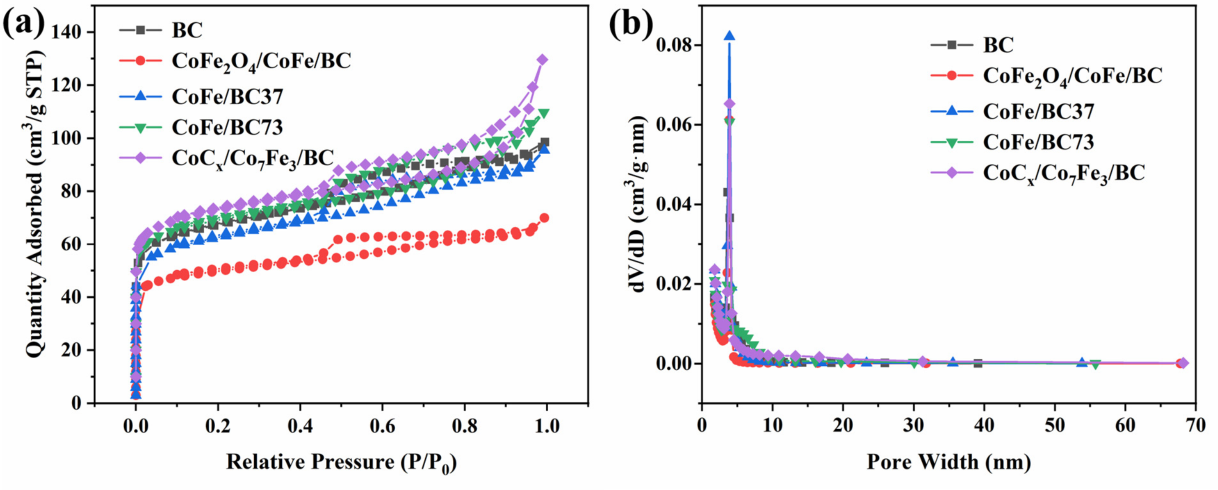

Figure S2. (a) N_2 adsorption-desorption isothermal curve and (b) pore size distribution curve of BC, $CoFe_2O_4$/CoFe/BC, CoFe/BC37, CoFe/BC73, and CoC_x/Co_7Fe_3/BC catalysts.

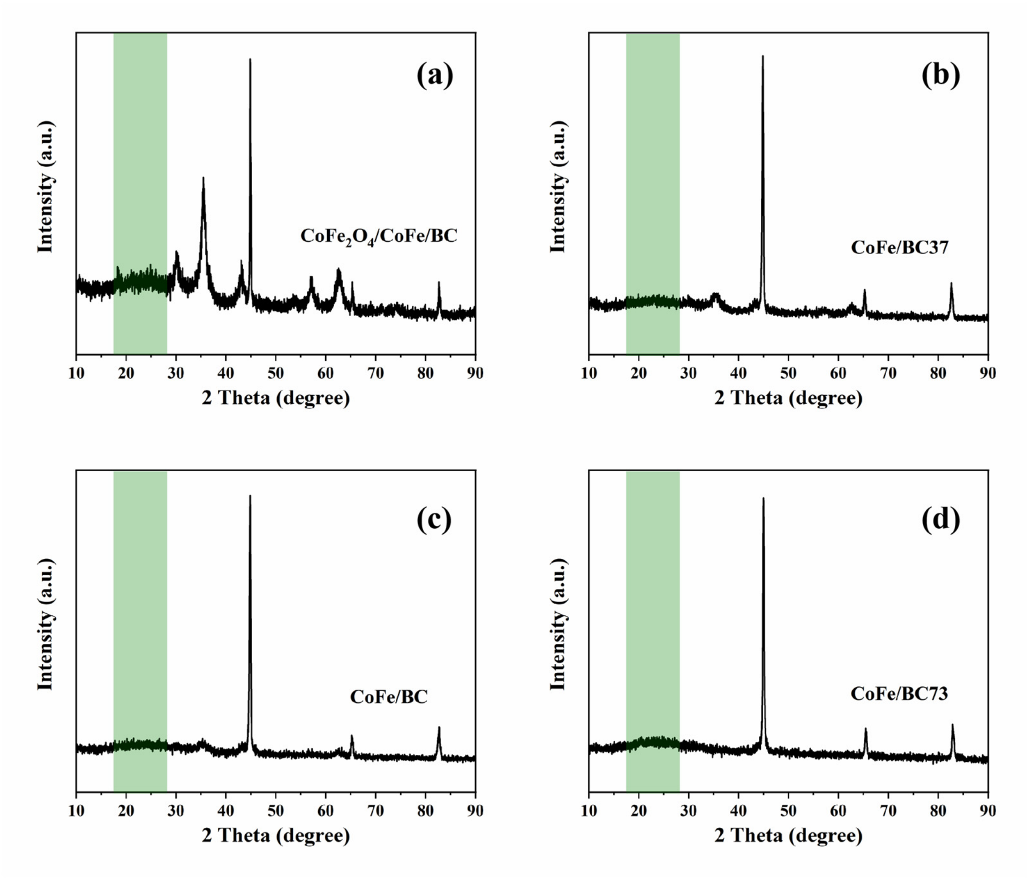

Figure S3. XRD patterns of (a) $CoFe_2O_4$/CoFe/BC, (b) CoFe/BC37, (c) CoFe/BC, and (d) CoFe/BC73.

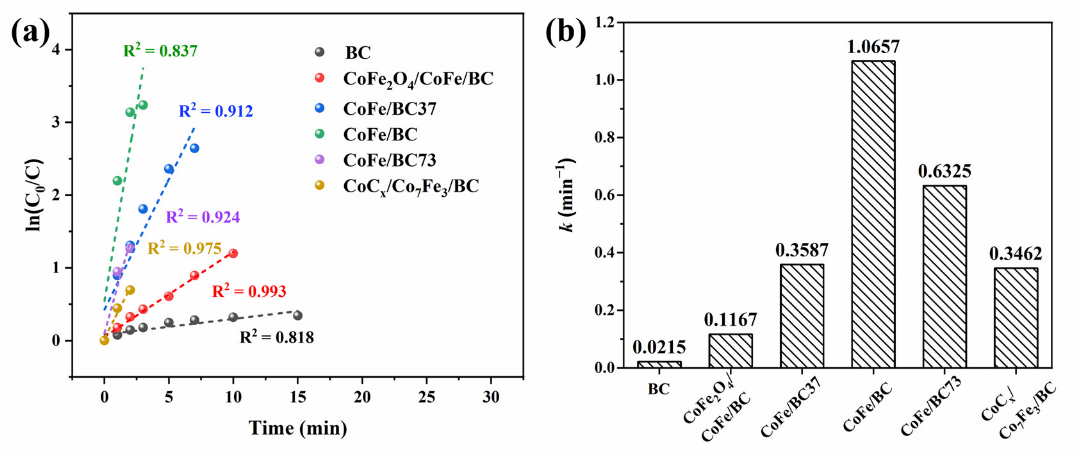

Figure S4. (a) Degradation kinetic curves and (b) the apparent rate constants k of BPF degradation in the different systems. Reaction condition: Catalyst = 0.05 g, BPF = 10 mg/L, PMS = 100 mg/L, T = 25 °C.

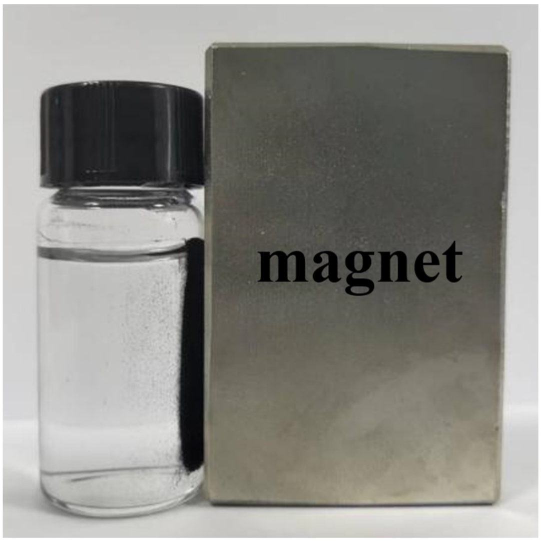

Figure S5. Photographs of the separation process of CoFe/BC catalyst with an external magnetic field.
